# Factors associated with success of vaginal birth after one caesarean section (VBAC) at three teaching hospitals in Addis Ababa, Ethiopia: a case control study

**DOI:** 10.1186/1471-2393-13-31

**Published:** 2013-02-01

**Authors:** Malede Birara, Yirgu Gebrehiwot

**Affiliations:** 1 Department of Obstetrics and genecology, Addis Ababa University, Addis Ababa, Ethiopia

## Abstract

**Background:**

Vaginal delivery after previous one cesarean section for a non recurring indication has been described by several authors as safe and having a success rate of 60–80%. Hence many centers are offering VBAC for candidates leaving the century old dictum of once cesarean always cesarean. But predicting success of VBAC after trial of labor (TOL) is still a difficult task due to the lack of a validated prediction tool. Studies on predictors of success are few and most of them conducted in developed countries and difficult to generalize. Therefore assessing factors associated with successful VBAC is very important to for counseling mothers while offering VBAC. The aim of this study was to assess factors associated with successful VBAC in three teaching Hospitals in Addis Ababa Ethiopia.

**Methods:**

A case control study was conducted to compare the factors associated with successful VBAC in teaching hospitals in Addis Ababa in one year period. The cases were those successfully delivered vaginally and the controls were those with failed VBAC and delivered by caesarean section. The sample size of the cases was 101vaginal deliveries and the controls were 103 failed VBAC patients which made the case to control ratio of 1:1.

**Result:**

In this study independent factors determining successful VBAC were, history of successful VBAC in the past, rupture of membrane at admission, and cervical dilatation of more than 3cm at admission. Presence of meconium, malposition and history of stillbirth were associated with failed VBAC. Factors like maternal age, past caesarean indications, inter delivery interval, and birth weight were not found to be significant determinants of success. The most common reason for repeat cesarean section for after trial of labor was labour dysfunction because of absence of a policy for augmentation on a scarred uterus in these hospitals.

**Conclusion:**

It is possible to prepare a decision tool on the success of VBAC by taking important past and present obstetric and reproductive performance history as predictor.

## Background

Caesarean delivery is an operation done to deliver a baby through an incision in the uterus. It is the most frequently performed surgical procedure worldwide [1]. Even though, variation exists in rates of caesarean delivery across countries; currently the rate ranges from 10% to 40% [1,2]. This high caesarean section rate has put burden on the economy of nations and individuals.

Previous caesarean section has been found to be the commonest cause of increased caesarean section rate in many parts of the world [1]. Because of increased risk of maternal complications with repeat caesarean section and safety of VBAC, trial of labour for selected group of patients with previous scar has become a preferred strategy [3].

In1988 ACOG recommended that, in the absence of a contraindication, a woman with one previous low-transverse cesarean delivery be counseled to attempt labor in a subsequent pregnancy [1,4].

Vaginal birth after cesarean section (VBAC) is associated with shorter maternal hospitalizations, less blood loss and fewer transfusions, fewer infections, and fewer thrombo-embolic events than cesarean delivery. Several reports have indicated that the absolute risk of uterine rupture attributable to a trial of labor is about 1 per 1000 [1-4].

A 60 to 80% success rate of vaginal birth after previous caesarean section has been reported by many authors if the primary caesarean was done for nonrecurring indications [2]. Some of the non recurring indications for caesarean section are: poor labour progress, foetal distress, placenta previa, transverse lie, breech presentation, oblique lie, pregnancy induced hypertension and twins [5].

The VBAC rate of hospitals in sub-Saharan Africa is between 37 to 97%. A Meta analysis done, in sub-Saharan countries showed a VBAC success rate of 63–75% [6].

There is considerable variation in the proportion of women who are offered and attempt VBAC across centres. British figures indicate that among women with a prior caesarean section, 33% will successfully achieve vaginal birth in the subsequent pregnancy. Again there was considerable variation across institutions, ranging from 6% to 64% [7].

One study in Lahore reported Successful vaginal delivery in 70% of the patients and repeat emergency caesarean section in 30% of the patients. The leading indications for the repeat caesarean sections were: failure to progress, fetal distress and scar tenderness. There were no maternal and foetal complications occurred. They concluded that VBAC is a safe practice [8].

Caesarean rate is increasing in Ethiopia because of the flourishing private hospitals in major towns. Even though teaching hospitals offer trial of labour for mothers with one scar, there is no study done which shows the rate of VBAC acceptance and success in Ethiopian Hospitals.

A study conducted in Brazil from1985 1995, the rate of TOL was found to be 11%. The factors significantly associated with vaginal delivery were monthly family income below 5-fold the Brazilian minimum monthly wage, reliance on the Brazilian national health system for healthcare, low maternal age, and first cesarean section indicated because of fetal breech or transverse presentation, or twin pregnancy [9].

Mother’s choice on mode of delivery is the most important single factor in offering trial of labour. Women’s expectations for birth and mode of birth preferences are influenced not only by knowledge of the potential benefits and risks but also demographic, obstetrical and social factors. This knowledge would help while counseling mothers for VBAC [4].

The crucial questions are how to reliably predict successful vaginal birth after Caesarean section, and how to determine and quantify the magnitude of the risk of failure that is acceptable to women. Many studies have addressed methods for identifying women at low and high risk of failure of an attempted vaginal birth after a prior caesarean but none of them have resulted in a validated result. Even those factors found to be associated with successful VBAC vary from centre to centre. Currently, therefore, there is no single validated tool which holds true for all to predict successful vaginal birth among women with a prior cesarean delivery.

The purpose of the present study was to identify maternal demographic, past and present obstetric determinants of successful VBAC in teaching hospitals in Ethiopia. This is of great help for physicians in the joint physician-patient decision while offering TOL.

## Methods

### Study area

The study was conducted in three hospitals in Addis Ababa, Ethiopia; namely, Tikur Anbesa (TAH) Hospital, St. Pauls (SPH) Hospital and Ghandi Memorial Hospital (GMH). These are teaching hospitals for Addis Ababa University which provide a 24 hr specialty care. Most of the deliveries and evaluations are made by residents with a specialist or faculty supervision. All hospitals offer trial of labour (TOL) with patient consent if the following conditions are fulfilled: the mother has one previous lower uterine segment scar, non recurring previous indications, singleton pregnancy, cephalic presentation, estimated fetal weight less than or equal to 4 kg, and no current indication for caesarean section.

### Study period

The study included mothers accepted a trial of labour (TOL) and delivered in the time period between May, 2009 and May, 2010.

#### Study design

A case control study was conducted to identify factors associated with successful VBAC among mothers with one previous cesarean section and offered trial of labor. Cases were all deliveries with only one previous scar, allowed VBAC according to the hospitals protocol, started labour spontaneously and delivered by vaginal route. Controls were those who delivered by caesarean section after trial of labour.

### Sample size and sampling technique

A double proportion sampling technique was used using EPI6 stat-calc. The sample size was calculated to get a minimum OR of 2 among cases over controls with 95% confidence interval; and power of 80%. The sample size was 204 of which 101 vaginal deliveries were taken as cases and 103 mothers who had failed VBAC and delivered by caesarean section were included in the control group.

The primary sources of the data were the admission log books at outpatient department (OPD) where the card numbers of patients admitted with previous caesarean scar was traced. Then those offered VBAC were identified from delivery log books and ward discharge summaries. Cases and controls were selected from the available charts in the study period until the minimum sample size was fulfilled.

Case ascertainment was done with clear inclusion and exclusion criteria. The inclusion criteria for the cases was patients with one previous lower uterine segment caesarean section scar who came with spontaneous labour or leakage of liquor, with no contra indication for VBAC and allowed to undergo trial of labour by the managing physician as documented on the patient chart and delivered through the vaginal route. The inclusion criteria for the controls were caesarean delivery after trial of labour.

### Data collection methods

Ethical clearance was obtained from Addis Ababa university department of OB-GYN research and publication committee and submitted to the hospital’s medical directors.

The data was collected from patients’ charts after tracing by patient’s number. Data was collected by residents after orientation on the data collection tool. The information was collected using a structured questionnaire which includes maternal socio demographic, past and present obstetric experience, mode of delivery and birth outcomes variables.

### Variables

Socio demographic variables: Maternal age, marital status, Parity, Gestational age, booking status, Address.

Past Obstetric variables: Indication for the primary C/S, inter delivery interval, Prior successful VBAC and Spontaneous vaginal delivery (SVD), history of still birth.

Current obstetric and foetal factors : Status of membrane at admission and duration rupture, presence of meconium, cervical dilatation at admission and position of the presenting part, duration of labour, birth weight and outcome of the baby.

### Analysis

Data was entered after checking completeness, cleaning and coding into computer SPSS version 15 software. A bivariate analysis was done using chi square test for difference of proportions between cases and controls for categorical variables and results showed in OR and respective confidence intervals. A multivariate analysis was run using logistic regression backward conditional model to control the confounding effect independent determinants.

## Results

The total number of mothers with one previous caesarean section who were offered trial of labour (TOL) and included in the study was 204. This number includes 101 cases who delivered vaginally after undergoing trial of labour. The 103 mothers for whom repeat caesarean section done after undergoing trail of labour were taken as controls

The exposure variables which were studied in this research were: demographic variables, reproductive history, past obstetric performance, and current obstetric variables like Status of membrane, Presence of meconium, cervical status, Duration of labour, Birth weight and outcome of the baby (see Table 1).

**Table 1:** Summary of exposure variables initially considered for the study


**I-Demographic Variables**	
Maternal age	
Marital status	
Address	
Education	
**II-Reproductive Variables**	
Parity	
ANC booking	
Gestational age	
Presence of co morbid medical illness	
**III-Past Obstetric variables**	
Indication for past caesarean section	
Inter delivery interval	
Presence of successful VBAC	
Past spontaneous vaginal delivery	
History of still birth	
**IV-Current obstetric variables**	
Membrane status at admission	
Cervical dilation at admission	
Duration of labour	
Presence of me conium	
Position	
Mode of delivery	
Birth weight	

In the analysis only those variables which are documented on the patients charts and logbooks were included. Therefore factors like, education, and medical illness were not documented on the data sources and taken off.

The majority of the study units were from Addis Ababa (93.1% of cases and 92% of controls) and few have come from nearby towns. Most of the mothers had their antenatal care at other health centres before they came for delivery to the hospitals.

The mean age of the cases and controls were 28.4 years and 28.6 years respectively. The mean parity and gestational age for cases was 2.4 and 39.8 weeks respectively. In this study significant association was not found between vaginal delivery and maternal age, gestational age, parity and place of ANC follow up.

It was not possible to know the indications for the past caesarean section for the Twenty percent of cases and ten percent of controls. Some of the indications for the last caesarean sections were fetal distress, malpresentations, big baby, failed induction and CPD. Of these indications fetal distress and failed induction were associated low success. The other indications were not found significant determinants.

Mothers who had experienced successful VBAC after the past caesarean section had a higher chance of success with significant statistical association. History of stillbirth in the past was found to be related with failed VBAC. We didn’t find significant relationship between success and past vaginal delivery before the previous caesarean section.

Regarding the current delivery, those mothers who were admitted after rupture of membrane, at active first stage of labor and having occipito-anterior position were having a higher chance of vaginal delivery which was found to be strongly statistically significant. Presence of meconium stained liquor and labour stay more than four hours after admission were associated with higher failure rate of VBAC which was also statistically significant. There was no association between the birth weight of the baby and success of VBAC. There were four still births among the cases but they were all intra uterine deaths.

Regarding the mode of delivery, 65 (64.5%) of the cases had spontaneous vaginal deliveries while the rest were instrumental deliveries. Among the controls the common indications for the cesarean section up on failed VBAC were slow progress (43.7%), arrest disorders (23.3%), none reassuring foetal heart pattern (NRFHRP) and CPD. There were 6 complications in the group of cases like perineal tear, episiotomy infection, and 16 in control group like wound infection, extensions and PPH. There was one cesarean hysterectomy.

Generally the independent factors found to be associated with successful VBAC by the multivariate analysis with logistic regression were history of stillbirth, history of successful VBAC, admission at active first stage of labor, and occipto-anterior positions (see Table 2).

**Table 2:** Bi variate analysis of cases and controls by selected present and past obstetric factors

	***Cases***	***Controls***	***CRUDE OR***	***95%CI***
***Maternal age***				
<*25*	*32*	*20*	***2***.***25***	***1***.***07***, ***4***.***73***
*26*-*30*	*42*	*59*	*1*	*1*
***Parity***				
*I*	*65*	*78*	*0*.*83*	*0*.*3*, *2*.*31*
*II*	*26*	*15*	*1*.*73*	*0*.*51*, *5*.*89*
*III and above*	*10*	*10*	*1*	
***Gestational age in weeks***				
<40	*31*	*32*	*1*.*03*	*0*.*56*, *1*.*97*
40	*18*	*20*	*1*.*13*	*0*.*53*, *2*.*39*
>40	*52*	*51*	*1*	
**Inter delivery interval**				
<2yrs	*17*	*9*	*1*	
*2*.*1*-*4*	*14*	*22*	*0*.*34*	*0*.*1*,*1*.*08*
*4*-*6*	*24*	*30*	*0*.*42*	*0*.*14*,*1*.*23*
>*6*	*46*	*42*	*0*.*58*	*0*.*21*,*1*.*58*
**History of stillbirth**				
yes	*10*	*21*	*1*	
No	*91*	*82*	***2***.***46***	***1***.***03***,***5***.***98***
**PAST C/S INDICATION**				
Known	*79*	*93*	***0***.***39***	***0***.***16***, ***0***.***92***
Unknown	*22*	*10*	*1*	
**Indication of the past cesarean section**				
Fetal distress	*16*	*25*	***0***.***29***	***0***.***1***,***0***.***86***
**Malpresentation**	*17*	*19*	*0*.*41*	*0*.*13*,*1*.*22*
Big baby	*10*	*11*	*0*.*41*	*0*.*11*,*1*.*40*
**Failed Induction**	*10*	*14*	*0*.*32*	*0*.*09*,*1*.*12*
CPD	*12*	*14*	*0*.*39*	*0*.*11*, *1*.*38*
Others	*14*	*10*	***0***.***64***	***0***.***18***, ***2***.***2***
Unknown	*22*	*10*	*1*	*1*
**Health of baby of past C/S**				
Live	*95*	*87*	***2***.***91***	***1***.***01***, ***8***.***77***
Dead	*6*	*16*	*1*	
**Prior successful VBAC in the past**				
Yes	*20*	*7*	***3***.***39***	***1***.***27***, ***9***.***34***
No	*81*	*96*	*1*	
**SVD before c/s**				
Yes	*22*	*18*	*1*.*32*	*0*.*62*, *2*.*79*
No	*79*	*85*	*1*	
**ROM at admission**				
Yes	*52*	*40*	*1*.*67*	*0*.*92*, *3*.*03*
No	*49*	*63*	*1*	
**Duration of ROM**				
<12hrs	*40*	*25*	***6***.***4***	***1***.***46***, ***32***.***1***
>12 hrs	*3*	*12*	*1*	
**Presence of Meconium**				
Yes	*11*	*27*	*1*	
No	*90*	*76*	***2***.***19***	***1***.***28***, ***6***.***72***
**Cervical Dilation at admission in cm**				
≤ 3	*28*	*70*	*1*	
>3	*73*	*33*	***5***.***53***	***2***.***91***,***10***.***56***
**Position of presenting part**				
OA	*38*	*21*	*1*	
OP/PT	*10*	*18*	***0***.***31***	***0***.***11***, ***0***.***86***
UK	*53*	*64*	***0***.***46***	***0***.***23***, ***0***.***92***
**Duration of labour after admission in Hours**				
<4 hrs	*56*	*42*	***1***	
>4 hrs	*45*	*61*	*1*.*81*	*1*, *3*.*28*
**Birth weight in grams**				
<2500	*7*	*3*	*5*.*83*	*0*.*48*, *93*.*2*
2500-4000	*92*	*95*	*2*.*42*	*0*.*4*, *18*.*5*
>4000	*2*	*5*	*1*	

## Discussion

This study was conducted with the main objective of identifying factors associated with successful vaginal delivery on mothers offered trial of labour after previous lower segment caesarean section. Significant Determinants found were history of still birth, history of successful VBAC, past indication of past C/S, presence of meconium, cervical dilatation at admission, rupture of membrane at admission and its duration and position of the presenting part. In this study parity, maternal age, gestational age, medical illness, HIV status, inter delivery interval, history of SVD, and birth weight were not found significant determinants.

The strengths of this study were the methodology, the use of inclusion and exclusion criteria. The case ascertainment and analyzing variables with less missing value has added to the strength.

There were some limitations in this study the study might be affected from small sample size effect because of errors made on the assumptions while calculating the sample size. There could also be a possibility of recall bias at reporting the inter delivery interval and indications of past cesarean section. Documentation on important variables, like occupational status, educational status, Apgar score, blood loss, post delivery hematocrit and medical illness was not good which has made these variables to be excluded from analysis.

Young maternal age and primiparity were associated with high success rate with vaginal delivery even though the bivariate analysis didn’t show that. Similar findings have also been reported by other authors [9]. Gestational age was not found a significant predictor of success in this study. There are reports which found that gestational age above 40 weeks is associated with poor success [4,9]. In our case the finding could also be confounded by high number of unknown dates and ascertainment of correct date was not possible.

History of still birth was one parameter which was associated with poor success in this study. This could be arising from the assumption that the cesarean route of delivery would give the mother a higher chance of having alive baby. Inter delivery interval was not associated with success of delivery. On other reports it was found that interval of less than 2 years was associated with poor success but not in our case [3]. This could be partly due to difficulty of case ascertainment and recall bias. Those mothers with unknown indications for past cesarean section have been found amazingly associated with high success. From known indications fetal distress, malpresentations and failed induction were associated with poor success. Breech presentation was not found significant determinant as opposed to other reports which consistently reported favoring successful vaginal delivery because of its less recurrence [3,6,7,9].

Prior successful VBAC was found to be associated with success which is similar to other reports [1,3,8,9]. Many authors reported history of prior spontaneous vaginal delivery as important determinant for success in VBAC. But our study failed to show that.

Status of membrane at admission was found to be important factor in predicting success of VBAC. Mothers admitted with rupture of membrane had a higher likelihood of success. But those whose liquor was stained with meconium have poor success.

The strongest factor determining success in this study was cervical dilatation at admission. Those who were admitted with cervical diameter greater 3 cm (Active first stage of labour) had a strong likelihood of vaginal delivery than those admitted at cervical diameter of less than or equal to 3 cm (latent first stage of labour). This is due to high frequency of false labour and slow progress in the latter. This is also supported by the finding that the mean cervical diameter at the time of cesarean section for failed VBAC group 4cm. The other important factor determining success was the position of the presenting part. Those having occipito anterior position were associated with higher success than those with occipito posterior and occipito transverse positions or unknown positions. There was no difference in the birth weight of both groups even though there are reports that macrocosmic babies have poor success. No difference in the neonatal outcome observed in both groups.

66% of repeat caesarean sections (66%) for the failed VBAC group were for reasons of slow progress of labour and arrest of cervical dilatations. This is because, oxytocin augmentation for scarred uterus is not allowed in these hospitals.

Generally the independent variables found to determine success of VBAC found with multivariate analysis were history of absence still birth, history of successful VBAC, cervical diameter at admission more than 3cm, and occipito anterior position of the presenting part (Table 3).

**Table 3:** Multivariate analysis of independent variables associated with successful VBAC

**Variable**	**Successful VBAC**	**Failed VBAC**	**CR OR **(**95% CI**)	**AOR **(**95% CI**)
No History of still birth	10/101	21/103	***2***.***46 ***(***1***.***03***,***5***.***98***)	2.54 (1.03,6.27)
Prior successful VBAC	20/101	7/103	***3***.***39 ***(***1***.***27***, ***9***.***34***)	3.40 (1.12, 9.48)
Cervical dilatation at admission > 3cm	28/101	70/103	***5***.***53 ***(***2***.***91***,***10***.***56***)	6.63 (3.36,13.01)
OP/OT positions	10/101	18/103	***0***.***31 ***(***0***.***11***, ***0***.***86***)	0.36 (0.14, 0.96)

## Conclusion

This study revealed that successful vaginal delivery after one previous cesarean scar was associated with past obstetrics performance and mainly to the current labor. The main determinants include history of stillbirth, history of successful VBAC in the past, rupture of membrane, absence of meconium, cervical stage of labor at admission, position of the presenting part, duration of labor, and knowledge of the previous indication for the past cesarean section.

VBAC is a safe practice as long as it is offered with proper selection of candidates with factors having a high success rate. Physicians need to be based on knowledge of factors having good outcome before counseling mothers so that failure rates decrease.

## Competing interests

We declare that there is no competing interest with anyone.

## Authors’ contributions

Both authors contributed equally to the study. MB developed the initial manuscript and YGH reviewed critically and approved for submission.

## Pre-publication history

The pre-publication history for this paper can be accessed here:

http://www.biomedcentral.com/1471-2393/13/31/prepub
